# Reducing short-acting beta-agonist overprescribing in asthma: lessons from a quality-improvement prescribing project in East London

**DOI:** 10.3399/BJGP.2021.0725

**Published:** 2022-08-23

**Authors:** Anna De Simoni, Hajar Hajmohammadi, Paul Pfeffer, Jim Cole, Chris Griffiths, Sally A Hull

**Affiliations:** Wolfson Institute of Population Health, Queen Mary University of London, London.; Wolfson Institute of Population Health, Queen Mary University of London, London.; Wolfson Institute of Population Health, Queen Mary University of London, London and Department of Respiratory Medicine, Barts Health NHS Trust, London.; Wolfson Institute of Population Health, Queen Mary University of London, London.; Wolfson Institute of Population Health, Queen Mary University of London, London.; Wolfson Institute of Population Health, Queen Mary University of London, London.

**Keywords:** asthma, electronic prescribing, primary health care

## Abstract

**Background:**

Excess prescription and use of short-acting beta-agonist (SABA) inhalers is associated with poor asthma control and increased risk of hospital admission.

**Aim:**

To quantify the prevalence and identify the predictors of SABA overprescribing.

**Design and setting:**

A cross-sectional study using anonymised clinical and prescribing data from the primary care records in three contiguous East London boroughs.

**Method:**

Primary care medical record data for patients aged 5–80 years, with ‘active’ asthma were extracted in February 2020. Explanatory variables included demography, asthma management, comorbidities, and prescriptions for asthma medications.

**Results:**

In the study population of 30 694 people with asthma, >25% (1995/7980), were prescribed ≥6 SABA inhalers in the previous year. A 10-fold variation between practices (<6% to 60%) was observed in the proportion of patients on ≥6 SABA inhalers/year. By converting both SABAs and inhaled corticosteroids (ICSs) to standard units the accuracy of comparisons was improved across different preparations. In total, >25% of those taking ≥6 SABAs/year were underusing ICSs, this rose to >80% (18 170/22 713), for those prescribed <6 SABAs/year. Prescription modality was a strong predictor of SABA overprescribing, with repeat dispensing strongly linked to SABA overprescribing (odds ratio 6.52, 95% confidence interval = 4.64 to 9.41). Increasing severity of asthma and multimorbidity were also independent predictors of SABA overprescribing.

**Conclusion:**

In this multi-ethnic population a fifth of practices demonstrate an overprescribing rate of <20% a year. Based on previous data, supporting practices to enable the SABA ≥12 group to reduce to 4–12 a year could potentially save up to 70% of asthma admissions a year within that group.

## INTRODUCTION

Frequent use of short-acting beta-agonists (SABAs) is a recognised marker of poor control^1^ and a potentially modifiable warning sign of impending serious asthma attacks^2^^–^^6^ and asthma death.^7^^–^^11^ Asthma control is defined as the extent to which the manifestations of asthma, commonly wheeze, shortness of breath, chest tightness, cough, and variable expiratory airflow limitation, can be observed in a patient on their current treatment.^12^^–^^14^ Control can be assessed by current symptoms and is indicative of future adverse outcomes.^1^^,^^12^ In East London, admission to hospital for acute asthma is 14% above the average for London, with hospital admissions rising from 1.3 to 7.5 per 100 asthma population as the number of SABA inhalers prescribed rises from 1–3 to >12 a year.^15^

In addition to the individual patient harm associated with excess prescription of SABAs, pressurised metered-dose inhalers are a major contributor to healthcare-associated carbon footprint, and thereby global warming.^16^ Hence, reducing SABA overprescribing is important both for individuals and for global health.

The dangers of SABA overprescribing have been highlighted in national (British Thoracic Society [BTS]^5^ and National Review of Asthma Deaths) and international^12^ (Global Initiative for Asthma [GINA]) guidance for many years. However, SABA overprescribing remains common across healthcare systems as highlighted in the recent SABINA studies.^6^ Notably, SABA overprescribing is more common in the UK than other European countries. Recent data show that SABA overprescribing (defined as collection of >2 SABA canisters/year) is associated with a dose-dependent increased risk of all-cause mortality and increased use of antidepressants, hypnotics, and sedatives, suggesting that those overprescribed SABA are a frailer patient group.^17^ Another recent study found that SABA overprescribing (≥3 prescribed inhalers/year) was prevalent across all GINA steps, which may indicate suboptimal asthma control,^18^ and concludes that further studies need to investigate the reasons behind SABA overprescribing, as well as effective interventions to reduce it.

Electronic alerts can reduce excessive prescribing of SABAs when delivered as part of a multicomponent intervention.^19^ In 2015, Asthma UK in conjunction with EMIS Web released a prescribing alert to highlight patients prescribed excessive SABAs. This activates if there are three prescriptions for SABA within a 3-month period. However, this assumes that only one device is issued per prescription and may underestimate SABA overprescribing over the longer term.

This current study forms the initial phase of a quality-improvement programme to reduce SABA overprescribing in East London. In this first step the aim is to:

- describe baseline data on SABA overprescribing in East London;
- standardise the calculation of prescription rates for SABA and inhaled corticosteroid (ICS) inhalers across formulations, taking into account the number of items on prescriptions;
- examine prescription modality as a previously underappreciated factor in SABA overprescribing.

**Table:** How this fits in

Excess prescription and use of short-acting beta-agonist (SABA) inhalers is associated with poor asthma control and increasing risk of hospital admission. This study found SABA overprescribing in >25% of the asthma population in East London, with a 10-fold variation in overprescribing rates among practices and a strong association with repeat dispensing (whereby prescriptions are issued automatically). Providing practices with tools to support the identification and management of high-risk patients based on prescribing records is warranted.

## METHOD

### Design and setting

This was a cross-sectional study using primary care electronic health data from 734 382 patients registered at all 117 practices in the East London boroughs of Newham, Tower Hamlets and Waltham Forest.

In the 2011 UK census, 55% of the population in these boroughs were recorded as being of minority multi-ethnicities,^20^ and the English indices of deprivation 2015 show that these localities fall into the top decile of the most socially deprived boroughs in England.^21^ The study population included patients aged 5–80 years registered at the practice for ≥1 year before data extraction in February 2020. All participants had a coded diagnosis of asthma and ≥1 prescription for inhaled asthma medication in the previous year.

### Data collection

Data were extracted on secure N3 terminals from EMIS Web. All data were anonymous and managed according to UK NHS information governance requirements. Demographic variables included age, sex, and self-reported ethnicity captured at registration with the practice or during routine consultations. Ethnic categories were based on the 18 categories of the UK 2011 census and were combined into four groups reflecting the study population.

Clinical measures included the latest value for smoking status, body mass index, and BTS asthma management step. To assess the burden of long-term conditions in the study population diagnostic data were extracted on 16 conditions that form part of the UK Quality and Outcomes Framework (QOF),^22^ using the earliest recorded diagnostic code before the start of the study, based on version 44 of the QOF business rule set. SNOMED codes (www.snomed.org) for chronic rhinitis and generalised anxiety were added to this. Prescribing data included all inhaled asthma medications and discrete courses of oral steroids in the previous year. All SABA inhalers were standardised to salbutamol 100 µg/dose (200-dose inhaler), and all inhaled steroid preparations were standardised using a method presented in Supplementary Information S1 and Supplementary Table S1.

Prescribing modalities included acute (provided by clinicians in response to an acute episode of illness) and automatic (automatic prescriptions are used for patients who will require medication without fail each month, for example, nursing home residents), repeat prescribing (regular long-term medications), and repeat dispensing (issued by a pharmacist from pre-authorised prescriptions for up to a year).

### Statistical analysis

The primary outcome measure was the proportion of asthma patients prescribed ≥6 standard SABA 100 µg/dose (200-dose salbutamol inhaler) equivalent inhalers in the previous 12 months.

All statistical analysis was undertaken in R (version 4.0.2). Both univariate and multivariate models were fitted. Sensitivity analyses were conducted to explore different groupings of comorbidities.

## RESULTS

Among the GP-registered population of 734 382, there were 30 694 people with asthma who fitted the study criteria (see Supplementary Figure S1). The characteristics of the study population are shown in Table 1, with the univariate odds for SABA overprescribing (≥6 SABA a year).

**Table 1.** Characteristics of the asthma study population

**Variable**	**Number**		**Univariate analysis^a^**		

	**SABA <6**	**SABA ≥6**	**OR**	**95% CI**	***P*-value**
**Age, years**					
Adult (18–60)	13 098	5970	Ref		
Child (5–17)	4828	1972	0.90	0.84 to 0.95	<0.01
Older adult (>60)	2686	2139	1.75	1.64 to 1.86	<0.01

**Sex**					
Male	9276	4651	Ref		
Female	11 336	5430	0.96	0.91 to 1.00	0.06

**Ethnicity**					
White	7048	3664	Ref		
Mixed	1052	474	0.87	0.77 to 0.97	0.02
Asian or Asian British	8572	4388	0.98	0.93 to 1.04	0.58
Black	2011	823	0.79	0.72 to 0.86	<0.01
Other	243	109	0.86	0.68 to 1.08	0.21
Not stated/unclassified	1686	624	0.71	0.64 to 0.79	<0.01

**IMD score**					
1 (least deprived)	2822	1306	Ref		
2	3756	1751	1.01	0.92 to 1.10	0.87
3	4526	2235	1.07	0.98 to 1.16	0.12
4	5199	2506	1.04	0.96 to 1.13	0.33
5 (most deprived)	4309	2283	1.14	1.05 to 1.24	<0.01

**Prescription type**					
Repeat	11 416	7895	Ref		
Acute and automatic^b^	9152	1955	0.31	0.29 to 0.33	<0.01
Repeat dispensed	44	231	7.59	5.55 to 10.63	<0.01

**Smoking**					
Never	15 345	6823	Ref		
Current	2417	1538	1.43	1.33 to 1.53	<0.01
Ex-smoker	2850	1720	1.36	1.27 to 1.45	<0.01

**BTS Asthma step**					
Step 1	3396	911	Ref		
Step 2	8415	4292	1.90	1.75 to 2.06	<0.01
Step 3	2029	1998	3.67	3.34 to 4.04	<0.01
Step 4 + step5	155	352	8.47	6.93 to 10.39	<0.01
Unknown	6617	2528	1.42	1.31 to 1.55	<0.01

**Oral steroid courses**					
Zero	17 622	7565	Ref		
1	2159	1373	1.48	1.38 to 1.59	<0.01
2	463	480	2.41	2.12 to 2.75	<0.01
≥3	368	663	4.20	3.69 to 4.78	<0.01

**Physical comorbidities**					
0	8263	3113	Ref		
1	8102	3618	1.19	1.12 to 1.25	<0.01
2–3	3618	2677	1.96	1.84 to 2.10	<0.01
≥4	629	673	2.84	2.53 to 3.19	<0.01

**Mental comorbidities**					
0	15 314	6703	Ref		
1	2926	1667	1.30	1.22 to 1.39	<0.01
2	2218	1528	1.57	1.47 to 1.69	<0.01
3	154	183	2.71	2.19 to 3.37	<0.01

**MPR category^c^**					
Reasonable use	2826	5109	Ref		
Zero use	4360	657	0.08	0.07 to 0.09	0.08
Underuse	13 314	2109	0.08	0.08 to 0.09	0.08
Overuse	112	2206	10.80	8.73 to 13.53	10.80

**Eosinophil count**					
No count	683	227	–		
<0.3	8707	4375	Ref		
≥0.3	6394	3507	1.2	1.06 to 1.18	<0.01

a *Univariate odds of using* ≥*6 SABA inhalers in the previous 12 months.*

b *The number of patients on automatic prescription was 285 for SABA* <*6 and 228 for SABA* ≥*6, respectively.*

c *MPR category: see details of MPR calculation in Supplementary Information S1. CI = confidence interval. IMD = Index of Multiple Deprivation. MPR = medication prescription refill. OR = odds ratio. Ref = reference. SABA = short-acting beta-agonist.*

Older adults had higher risks of SABA overprescribing, as did those with increasing numbers of physical and mental comorbidities. Prescription modality — in particular repeat dispensing — was also strongly associated with the risk of SABA overprescribing. In keeping with more severe disease, there was a significant association between higher asthma management step and increased SABA prescriptions. Furthermore, consistent with airways inflammation as a risk factor for uncontrolled asthma, there was a significant association between eosinophilia (eosinophil count ≥0.3) and SABA overprescribing.

Children were found to have the lowest risk of SABA overprescribing; hence subsequent analyses have been undertaken in the adult population only.

**Figure 1.**
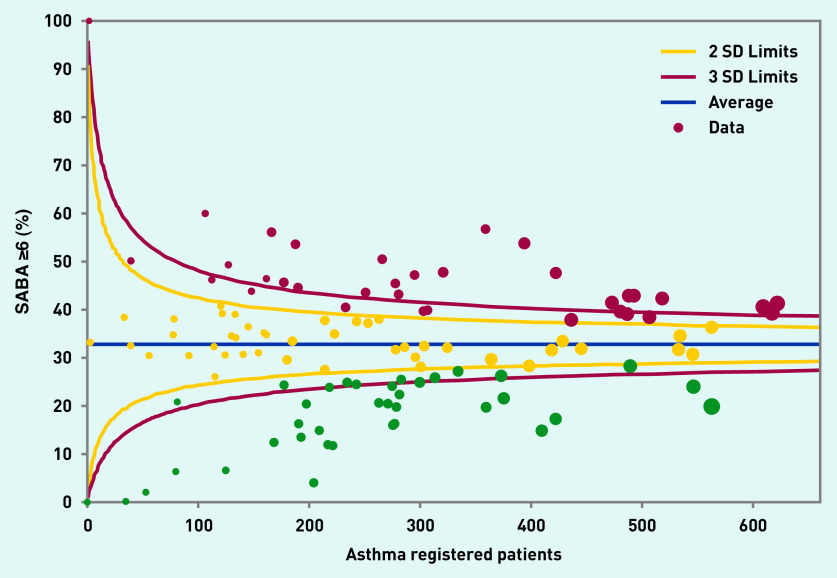
*Funnel plot illustrating practice variation in the proportion of patients with asthma prescribed ≥6 SABA inhalers in the previous 12 months (y-axis). Data from 117 practices in East London, February 2020. Green dots represent practices and colours are used according to traffic light system: in red practices with the highest percentage of patients overprescribed SABA*. *The y-axis is the % of asthma patients prescribed ≥6 SABA. The x-axis shows the size of the asthma population in the practice (larger dots represent practices with a larger asthma population). SABA = short-acting beta-agonist.*

Analysis by GP practice (see Figure 1) shows a 10-fold variation between practices in the percentage of asthma patients prescribed ≥6 SABA in the previous 12 months (range <6% to 60%). This variability was distributed uniformly across practices of all sizes. About one-fifth of practices had an overprescribing rate of <20% (23/117).

To identify factors associated with SABA overprescribing among adults a multivariate model was used (Table 2). Severity of asthma, as measured by asthma step and by number of oral steroid prescriptions, were both major independent predictors of SABA overprescribing.

**Table 2.** Logistic regression model showing the predictors of SABA overprescribing (≥6 SABA in the previous 12 months) for adults with asthma

**Variables**	**Number**		**Multivariate logistic model^a,b^**		

	**SABA <6**	**SABA ≥6**	**OR**	**95% CI**	***P*-value**
**Age**					
Adult	13 098	5970			
Older adult (>60)	2686	2139	1.34	1.24 to 1.45	<0.01

**Sex**					
Male	6392	3430			
Female	9393	4679	0.85	0.90 to 0.80	<0.01

**Ethnicity**					
White	6066	3297			
Mixed	6082	3297	0.87	0.75 to 1.00	0.39
Asian or Asian British	1568	655	1.13	1.05 to 1.21	0.02
Black	778	352	0.94	0.84 to 1.06	0.15
Other	186	81	1.01	0.76 to 1.36	0.03
Not stated/unclassified	1104	427	0.84	0.74 to 0.96	0.03

**Prescription type**					
Repeat	8671	6392			
Acute and automatic	7074	1513	0.29	0.27 to 0.31	<0.01
Repeat dispensed	39	204	6.52	4.64 to 9.41	<0.01

**Smoking**					
Never	10 512	4848			
Current	2395	1524	1.51	1.38 to 1.63	<0.01
Ex-smoker	2840	1716	1.21	1.13 to 1.32	<0.01

**Asthma step**					
Step 1	2485	670			
Step 2	6218	3289	1.79	1.62 to 1.97	<0.01
Step 3	1864	1826	2.87	2.56 to 3.21	<0.01
Step 4+5	136	323	4.99	3.96 to 6.30	<0.01
Unknown	5082	2001	1.19	1.05 to 1.35	<0.01

**Oral steroid**					
Zero	13 188	5955			
1	1851	1139	1.35	1.21 to 1.45	<0.01
2	392	412	2.67	2.48 to 2.71	<0.01
≥3	354	603	2.90	2.46 to 3.31	<0.01

**Physical comorbidities**					
0	5401	2044			
1	6307	2802	1.07	1.00 to 1.16	0.05
2–3	3448	2591	1.58	1.47 to 1.73	<0.01
≥4	628	672	1.86	1.61 to 2.13	<0.01

**Mental comorbidities**					
0	10 630	4795			
1	2796	1612	1.19	1.10 to 1.28	<0.01
2	2204	1519	1.32	1.22 to 1.44	<0.01
3	154	183	2.29	1.80 to 2.91	<0.01

a *This model is adjusted by Index of Multiple Deprivation score.*

b *This model is a mixed-effect multivariate logistic regression, which includes clustering based on the practice (Newham, Tower Hamlet and Waltham Forest). The adjusted interclass correlation coefficient of this variable is 0.006, which is negligible. CI = confidence interval. OR = odds ratio. SABA = short-acting beta-agonist.*

The multivariate analysis confirmed that older adults (odds ratio [OR] 1.34, 95% confidence interval [CI] = 1.24 to 1.45), smoking, and increasing number of both physical and mental comorbidities were independent predictors of SABA overprescribing.

This study has also confirmed that prescribing modality and in particular repeat dispensing (where repeat medications are managed by community pharmacists) was strongly associated with excess SABA use. Repeat dispensing compared with repeat prescribing had an OR of 6.52 (95% CI = 4.64 to 9.41). Although patients on repeat dispensing only represent about 1% of the population with asthma (243/23 893 in Table 2), 84% of them (204/243) were prescribed ≥6 SABA/year.

Previous studies have suggested that overprescribing of SABAs is associated with underprescribing of ICSs. Using the more precise measures of the medication prescription refill (MPR) % for preventer ICS/ICS-LABA (long-acting beta 2-agonists) inhalers, which compares prescribed ICS against standard expected use, in the current study it was possible to categorise ICS use for each patient for the previous 12 months as: MPR underprescribing (<50%); MPR expected use (≥50% to 120%) and MPR overprescribing (>120%) (see Table 3, Supplementary Information S1, and Supplementary Table S1 for further details).

**Table 3.** ICS categorisation based on MPR percentage

**Description**	**MPR %**
• Overprescribing	≥120
• Expected prescribing	≥50 to 120
• Underprescribing	<50

*See Supplementary Information S1 for methods. ICS = inhaled corticosteroid. MPR = medication prescription refill.*

**Figure 2.**
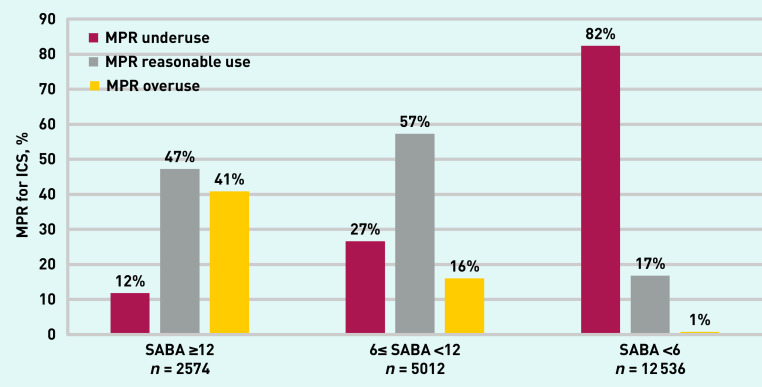
*MPR rate for ICS for the different categories of SABA prescribing (SABAs ≥12, 6≤ SABA <12, and SABA <6) among adult patients prescribed ICS in the previous year. See details of MPR calculation in Supplementary Information S1. ICS = inhaled corticosteroid. MPR = medication prescription refill. SABA = short-acting beta-agonist.*

MPR use across the range of SABA prescribing in the previous year was examined. Figure 2 shows the fall in higher-than-expected ICS use (labelled ‘MPR overprescribing’) in parallel with the fall in number of SABA inhalers issued. It also shows that among patients prescribed between 6 and 11 SABA inhalers/year (likely a population with less controlled asthma) over a quarter of patients were issued fewer ICS prescriptions than expected (‘MPR underprescribing’). More than 80% (18 170/22 713), were underprescribed ICSs in the group of patients with SABA prescriptions <6 a year (likely a population with milder, better controlled asthma). Patients prescribed between 6 and 11 SABA inhalers a year and underprescribed MPR are a target group to focus on to reduce SABA overprescribing. Some may have significant asthma but inadequate preventive treatment that if improved will reduce their SABA prescriptions, others may need a review of their asthma diagnosis.

To calculate how many hospital admissions could be avoided by improving asthma management and hence reducing SABA overprescribing, the results of a previous publication^15^ based in the same population were used. In the current study, the hospital admission rate for SABAs ≥12 was 7.5% a year. As an estimate, by enabling a reduction of SABA prescribing for this group to between 4 and 12 SABAs (associated with a hospital admission rate of 2.3% a year), there is potential to avoid up to 70% of hospital admissions in this group.

## DISCUSSION

### Summary

Rates of SABA overprescribing remain high in this multi-ethnic, deprived urban population, with significant variation among practices. About one-fifth of practices achieved a SABA overprescribing rate of <20% (23/117).

By converting both SABAs and ICSs to standard units in this study it was possible to make more accurate comparisons across prescribed medications. Among adult patients prescribed between 6 and 12 SABA inhalers/year (representing a population with less controlled asthma) over a quarter of patients were issued less ICS prescriptions than expected. Working with these patients to improve regular preventer use should be an early target to reduce SABA overprescribing by improving their asthma control and reducing breakthrough asthma symptoms for which they may take reliever medication.^23^

Logistic regression analysis shows that alongside markers of asthma severity, multimorbidity and the type of prescription are important independent predictors of SABA overprescribing.

### Strengths and limitations

The study results are based on patients with asthma from a population of almost one million GP-registered patients in East London using prescribing data for a full year before data extraction. Results from this study will be generalisable to other multi-ethnic inner urban populations in the UK. With >93% ethnicity recording, it was possible to explore the contribution of ethnicity and deprivation alongside other established risk factors for SABA prescribing.

The asthma population in this study is based on diagnostic codes and asthma medication prescribed in the previous year, however, asthma overdiagnosis is known to occur in primary care^24^ hence some patients may have alternative diagnoses. The study measures prescriptions issued rather than medication taken. Receiving a prescription does not necessarily mean that the prescription was filled and used, and it is possible that ‘stock piling’ inhalers may inflate estimates of SABA overprescribing. It will also contribute to medication waste. The MPR calculations, comparing prescribed medication against standard expected use, are based on typical regimens for specific inhalers (See Supplementary Table S1), not the actual prescribed dosing frequency for individuals.

### Comparison with existing literature

The results of the current study show that rates of SABA overprescribing are consistent with those reported in a recent Swedish study^17^ with patients aged 12–45 years and a German study of >12-year-olds,^18^ which found that about a third of patients with asthma are prescribed >3 SABAs/year. In common with the current study they developed a standard measure for ICSs (low, medium, and high dose), using budesonide equivalent doses.^17^ Consistent with the current findings, the proportion of patients with SABA overprescribing was higher in patients at GINA steps 3–5 compared with those at GINA step 1 or 2, a sign of poor asthma control in patients with severe asthma.^17^ This poor control was observed despite patients receiving ICSs/LABA. Indeed, at GINA steps 3–5, the risk of SABA overprescribing was highest in patients using ICSs/LABA. A recent multinational qualitative study^25^ aimed at identifying drivers of patients’ reliance on SABAs in asthma revealed that patients can have a strong emotional attachment to SABA relievers, driven by their efficacy and success in quickly alleviating symptoms. Moreover, some patients typically do not understand that the frequent use of SABAs indicates poor asthma control, whereas others have a misperception of ICSs, which could lead to a delay in escalation and poor adherence. Experiencing severe exacerbations can improve adherence to ICSs, but only temporarily in many cases.

Further, some adolescents and young adults who are high users of SABAs adapt poorly to having asthma and have poor asthma control: overprescribing of SABAs is a convenient way to enable them to live their lives.^26^

### Implications for practice

The wide variation in SABA overprescribing rates between practices in the same localities suggest there is potential to reduce overprescribing rates in higher-prescribing practices. Sharing data on comparative prescribing rates between practices encourages reflection and the development of shared strategies for the reduction of overprescribing.^27^

Providing practices with software tools to identify patients with asthma at high risk of hospital admission based on prescribing records is warranted. Such tools should be integrated with the software used by primary care teams, and enable the automatic flagging of patients overprescribed SABAs. Engaging practice teams to deliver systematic, structured asthma reviews is key to the optimisation of asthma management and prescribing.

Tackling SABA overreliance aligns with both the drive to improve asthma control and the drive to reduce the environmental impact of asthma care. This context offers an opportunity to significantly reduce the carbon footprint of the NHS.

The current study results highlight the importance of general practice teams working effectively with pharmacists to ensure a shared understanding on access to SABA medications. In some cases this may require removing SABA medications from repeat dispensing.

Improving asthma management, by adequate preventer treatment, education, and regular support can translate into a reduction in acute hospital admissions. If all practices were enabled to support patients prescribed >12 SABAs a year to reduce to 4–12 a year there is potential to reduce up to 70% of asthma admissions a year for this group.

## Data

All data relevant to the study are included in the article.
